# Genetics of *PLCG2* expression and splicing relative to Alzheimer’s disease risk

**DOI:** 10.21203/rs.3.rs-6735123/v1

**Published:** 2025-06-19

**Authors:** Andrew K. Turner, Kennedy Dotson, Qi Qiao, Kailey Cain, James F. Simpson, David W. Fardo, Steven Estus

**Affiliations:** University of Kentucky; University of Kentucky; University of Kentucky; University of Kentucky; University of Kentucky; University of Kentucky; University of Kentucky

**Keywords:** Alzheimer’s disease, RNA splicing, genetics, PLCG2

## Abstract

**Background:**

PLCG2 is associated with the risk of Alzheimer’s disease (AD) through a rare missense polymorphism, rs72824905 (P522R) as well as a common variant, rs12445675, within a long non-coding RNA adjacent to *PLCG2*. Elucidating the impact of genetics on PLCG2 expression and splicing will provide insights into the role of PLCG2 in AD risk and, potentially, treatments that might reduce AD risk.

**Objective:**

To evaluate *PLCG2* expression and splicing as a function of AD genetics.

**Methods:**

*PLCG2* isoform expression was detected by PCR and quantified by qPCR in AD and non-AD brain samples and in blood buffy coat samples. The function of a genetic variant, rs107164, was tested by using a minigene approach with both alleles in murine BV-2 microglial cells. The impact of ectopic splicing factor expression on PLCG2 minigene splicing was also tested in BV-2 cells. The extent that endogenous levels of a novel *PLCG2* mRNA isoform lacking 65 bp within exon 28 (D65-PLCG2) were affected by nonsense mediated decay (NMD) was determined by using cycloheximide *in vitro*. Lastly, whether *D65-PLCG2* manifested a Ca + 2 response similar to *PLCG2* was tested by comparing D65-PLCG2-GFP and PLCG2-GFP fusion proteins in transfected HEK293 cells.

**Results:**

We report *PLCG2* isoforms that include (i) a transcript that replaces *PLCG2* exon 1 with sequence from an adjacent long noncoding (LNC) RNA (*LNC-PLCG2*) and (ii) a transcript that lacks 65 bp from the beginning of exon 28 (*D65-PLCG2*). The ratio of *LNC-PLCG2* to canonical *PLCG2* was associated with rs12445675 genotype in both human brain and buffy coat samples. The proportion of *PLCG2* expressed as *D65-PLCG2* was increased by the T allele of rs1071644, a T/C SNP within the 65bp variably spliced portion of exon 28. This SNP was demonstrated to be functional in a minigene splicing assay. Moreover, the rs1071644-T allele was found to be associated with increased AD risk, independent of rs72824905 (P522R) and rs12445675. *D65-PLCG2* was susceptible to nonsense mediated RNA decay. D65-PLCG2 was not responsive to Ca^+ 2^ in a fashion similar to that observed for PLCG2. Hence, the rs1071644-T allele appears to increase AD risk by increasing the proportion of *PLCG2* expressed as *D65-PLCG2*, representing a loss of PLCG2 function.

**Conclusions:**

We report that two AD genetic risk factors, rs12445675 and rs1071644, affect AD risk by impacting the *LNC-PLCG2* to *PLCG2* ratio and *PLCG2* exon 28 splicing, respectively.

## 1. Introduction

Twin and family studies indicate that genetics underlies the majority of Alzheimer’s disease (AD) risk (Gatz et al., 2006). Elucidating the mechanisms underlying these AD risk factors will provide insights into disease mechanisms and may facilitate the identification of novel therapeutics. Two primary single nucleotide polymorphisms (SNP)s implicate *PLCG2* in AD. First, a rare missense SNP, rs72824905 (P522R), reduces AD risk (Dalmasso et al., 2019; Kleineidam et al., 2020; Magno et al., 2019; Olive et al., 2020; Sims et al., 2017; Strickland et al., 2020; van der Lee et al., 2019) and appears a mild hypermorph in *PLCG2* function (Magno et al., 2019; Solomon et al., 2022; Takalo et al., 2020). Second, a common SNP upstream of *PLCG2*, rs12446759, within the LNC adjacent to *PLCG2*, also reduces AD risk and has unclear actions on *PLCG2* (Bellenguez et al., 2022; Dalmasso et al., 2019; Jansen et al., 2019; Kleineidam et al., 2020; Magno et al., 2019; Olive et al., 2020; Sims et al., 2017; Strickland et al., 2020; van der Lee et al., 2019).

Prototypic *PLCG2* contains 33 exons with translation beginning from an ATG within exon 2. *PLCG2* encodes a phospholipase and, within the brain, is primarily expressed by microglia (Sims et al., 2017). While our understanding of microglial function in AD is still evolving, microglia are currently thought to be protective early in the AD process as they contribute to amyloid-beta clearance and possibly deleterious late in AD as their activation and associated inflammation may promote neurofibrillary tangle formation and cognitive decline (reviewed in (Leng & Edison, 2021; Sudwarts & Thinakaran, 2023)). Within microglia, *PLCG2* is activated by increased cytosolic Ca^+ 2^ to cleave 1-phosphatidyl-1D-myo-inositol 4,5-bisphosphate (PIP2) to 1D-myo-inositol 1,4,5-trisphosphate (IP3) and diacylglycerol (DAG), which are key secondary messengers. As such, *PLCG2* transduces signals from cell surface receptors such as TREM2, which has itself been associated with AD risk (Jay et al., 2017; Shi & Holtzman, 2018).

Here, to gain insight into the role of *PLCG2* expression and splicing in AD, we report an isoform that begins in an LNC RNA adjacent to PLCG2 (*LNC-PLCG2*) and an isoform that lacks the initial 65bp of exon 28 (*D65-PLCG2*). The ratio of the *LNC-PLCG2* to prototypic *PLCG2* was associated with rs12446759. The proportion of *PLCG2* expressed as *D65-PLCG2* was associated with rs1071644, a SNP within the skipped portion of exon 28. This SNP was found to be functional in modulating the proportion of *PLCG2* expressed as *D65-PLCG2* in minigene assays. Moreover, this SNP was associated with AD risk independent of either P522R or rs12445675. This aberrant *PLCG2* splicing was correctable by ectopic expression of select splicing factors by using a minigene model. The loss of 65bp from exon 28 results in a premature termination codon such that this isoform encodes a PLCG2 lacking its carboxyl sequence, which includes a Ca^+ 2^ binding domain. *D65-PLCG2* was susceptible to nonsense mediated RNA decay and produced a protein lacking the Ca^+ 2^ response observed in PLCG2. Overall, we report that rs12445675 and rs1071644 affect AD risk by impacting the *LNC-PLCG2* to *PLCG2* ratio and *PLCG2* exon 28 splicing, respectively.

## 2. Materials and Methods

### Human Brain DNA and cDNA

The genomic DNA and cDNA brain samples used have been described extensively (Shaw et al., 2021; Shaw et al., 2022; Zajac et al., 2023). In short, anterior cingulate samples were provided by the University of Kentucky AD Research Center and purified for RNA and genomic DNA, with the RNA converted to cDNA as described (Shaw et al., 2021; Shaw et al., 2022; Zajac et al., 2023). The National Institute on Aging-Reagan Institute criteria for neuropathological diagnosis of AD based on amyloid and tau deposits were used as a measure of AD neuropathology. Scores of “intermediate likelihood” or below were defined as low AD neuropathology and scores of “high likelihood” were defined as high AD neuropathology (reviewed in (Nelson et al., 2009)). The samples for this study were from 25 brains with low neuropathology and 24 brains with high AD neuropathology, with an average age at death of the brain donors of 82.0 ± 9.1 (mean ± SD, n = 25) and 82.0 ± 6.5 (n = 24) years, respectively. The post-mortem interval for the high neuropathology and low neuropathology samples was 3.4 ± 0.6 and 2.8 ± 0.9 hours, respectively. Buffy coat samples were obtained from cognitively intact individuals, including 28 men with an average age of 72.6 ± 1.1 years and 29 women, average age of 71.0 ± 1.0 years. These samples were stored at −80C until RNA was purified by sequential TRIzol phase separation and RNeasy spin columns, as described (Haynes, 2015). RNA was converted to cDNA by using Superscript IV, as described by the manufacturer (Thermo). DNA was prepared from these samples by using Qiamp Blood DNA minikits, as directed by the manufacturer (Qiagen).

### PCR

Genomic DNA samples were genotyped by using TaqMan Genotyping (Thermo) as directed by the manufacturer: initial denaturation at 95°C for 10 min, and PCR cycling at 95°C, 15s; 60°C, 1 min; 40 cycles. For the initial identification of *LNC-PLCG2* isoforms, polymerase chain reaction (PCR) was performed with primers corresponding to sequences within the *LNC* exon 1 (5’ TGCAGTCACACAGCCAACTT) and *PLCG2* exon 2 (GGGAGAAGGAAGGAATCGGG). To detect novel *PLCG2* splice variants in human brain, PCRs were performed on cDNA samples corresponding to 30 ng of RNA with primers corresponding to sequences within: exon 1 (5’- CCGGAGCCCAAACCCG) and exon 6 (5’- GATCAGGGGCAAGATGGTCT), exon 5 (5’- CTGTGGATCAAACCAGAAGAAAC) and exon 10 (5’- ACTTTGTCATCCGCTCACGG), exon 9 (5’- ATGCCTCTGCTGTTTACCTG) and exon 14 (5’- TGTGACGCTGTTGCTCCA), exon 13 (5’- GCCATCAAAGACCACGCCTT) and exon 17 (5’- ACTTCTCGGCACTCGTCCT), exon 16 (5’- CACTACTGCGCCATTGCY) and exon 19 (5’- CAGCATGTCCTCTGCCTCTC), exon 18 (5’- CTATGCCCTCATCCAGCACT) and exon 22 (5’- ATCGCTTCGCTTGGCTTTGT), exon 20 (5’- AAGCATTGTCGCATCAACCG) and exon 26 (5’- TCCACATACATTCTGCTGACG), exon 25 (5’- TGAGTGGTTTCAGAGCATCCG) and exon 29 (5’- AGGGACAGGCAATACTTCGTC), exon 28 (5’- TATGACCCGATGCCACCCG) and exon 32 (5’- TTCAGTTCTTCTTGCCGCCT), and exon 31 (5’- TCCCTCCTGGTTTTCTGTGAG) and exon 33 (5’- ACTTGCTGTTGCTGACTCTCTT). After 30 cycles with Q5 DNA Polymerase (New England Biolabs), PCR products were separated by electrophoresis using a 10% polyacrylamide gel and visualized with SYBRgold staining. Isoforms were excised and eluted from the gel, reamplified with appropriate primers and sequenced (ACGT, Inc).

### qPCR

To quantify *PLCG2* isoforms, a series of qPCR assays were performed as described previously (Shaw et al., 2021; Shaw et al., 2022; Zajac et al., 2023). For each assay, copy numbers present in cDNA samples were determined relative to standard curves that were executed in parallel (Shaw et al., 2021; Shaw et al., 2022; Zajac et al., 2023). The *LNC-PLCG2* was quantified using a forward primer corresponding to the *LNC* exon 2 (5’- CAGGCGTCAGGAAAAGAACA) and a reverse primer corresponding to *PLCG2* exon 2 (5’- GGGAGAAGGAAGGAATCGGG). Results were compared with those for canonical *PLCG2* which used a forward primer corresponding to *PLCG2* exon 1 (5’- CGGAGGGCGTGAGCG) and an exon 2 reverse primer (5’-CGGGGGTGGACTTGCG). Total *PLCG2* was quantified using a forward primer corresponding to constitutively present sequence in exon 28 (5’-GAATCACGCATTGTTTTCTCTCA) and in reverse primer corresponding to sequence in exon 29 (5’- AGGGACAGGCAATACTTCGTC). The *D65-PLCG2* was quantified with the same reverse exon 29 primer and a forward primer corresponding to the novel junction between exons 27 and 28 created by the loss of the first 65 bp in exon 28 (5’- TTCCAGACGGCAGCCTG). The PCR cycling conditions for all qPCR were as follows: 95°C, 2 min; 95°C, 15 s, 60°C, 15 s, 72°C, 30 s, 40 cycles.

### Genetic Association Analyses

Genetic data from the Alzheimer’s Disease Genetics Consortium (ADGC) were obtained and linked to participant data from the National Alzheimer’s Coordinating Center (NACC) as described previously (Katsumata et al., 2022). The variable defining presumptive etiologic diagnosis of the cognitive disorder - Alzheimer’s disease (NACCALZD) was used to define AD cases and controls. Participants with mild cognitive impairment (MCI) or with impairment and no Alzheimer’s disease etiologic diagnosis as defined in NACCUDSD were excluded, resulting in 8230 controls and 8215 AD cases. Logistic regression assuming an additive mode of inheritance and adjusting for sex, age, and 10 PCs was conducted in PLINK 2.0 (Chang et al., 2015; Purcell & Chang).

### PLCG2 Minigene

To generate *PLCG2* minigenes consisting of exon 27, exon 28, exon 29, and their intervening introns, primers corresponding to sequence in exon 27 (5’- CATCATCAGACAGAAGCCCGT) and exon 29 (5’- GTCATACTCGGCTCCACAGA) were used to PCR-amplify (Q5, NEB) genomic DNA from individuals homozygous for each allele of rs1071644. PCR products were separated by electrophoresis on a 1% agarose gel, excised, eluted, and then TA-cloned into pcDNA3.1 (Thermo). Clone sequences were confirmed via sequencing (Plasmidsaurus). In addition to rs1071644, clones also differed for rs4611451, rs12596299, rs4888191, rs488192 and rs4369659. To determine whether rs1071644 was indeed the functional SNP, the clone with rs1071644-C was mutated to rs1071644-T with a Quikchange Site Directed Mutagenesis kit (Agilent) as directed by the manufacturer by using HPLC purified primers 5’- CTCAATTTCCAGACGGCAAAGGGCAATTCTGCAGAT-3’ and 5’- ATCTGCAGAATTGCCCTTTGCCGTCTGGAAATTGAG-3. The resulting clone was then sequenced (Plasmidsaurus) to confirm mutagenesis at only the rs1071644 site. These three clones were then transfected in triplicate into murine microglial BV-2 cells using Lipofectamine 3000 reagent (Thermo), as directed by the manufacturer. Cells were incubated for 24 hours and RNA purified with RNeasy (Qiagen) following manufacturer’s directions. RNA was converted into cDNA via reverse transcription by using random primers and Superscript IV Reverse Transcriptase (Thermo). *PLCG2* exon 28 splicing was then quantified by qPCR by using a reverse primer that corresponded to vector -derived transcript sequence (5’ AGACCGAGGAGAGGGTTAGG) and forward primers corresponding to either exon 27 (5’- CATCATCAGACAGAAGCCCGT) or to the unique sequence corresponding to the exon-27-exon 28 junction observed in *D65-PLCG2* (5’-TTCCAGACGGCAGCCTG).

### Nonsense Mediated Decay

To determine if *D65-PLCG2* undergoes NMD, the U937 human cell line, which constitutively expresses *PLCG2*, was treated with cycloheximide (CHX) or a DMSO solvent control as described previously (Zajac et al., 2023). Briefly, U937 cells maintained in RPMI 1640 with HEPES (Invitrogen 42401–018), 10% v/v fetal calf serum (characterized, low lipopolysaccharide), 50 U/mL penicillin and 50 μg/mL streptomycin were plated in 0.9 mL media in a 24-well plate. CHX was dissolved in ethanol at 50 mg/mL and cells were treated in triplicate with either CHX (final concentration of 50μg/ml) or ethanol (0.1%). After 1, 3, 5, or 8 hours, cells were centrifuged and RNA purified using RNeasy according to the manufacturer’s instructions (Qiagen). RNA was converted to cDNA by using random hexamers and SuperScript IV (Thermo, Waltham, MA USA). *D65-PLCG2* and *PLCG2* were visualized by PCR followed by gel electrophoresis and SYBR-gold staining and quantified by qPCR, as described above.

### Splicing Factors

To determine if the *D65-PLCG2* isoform is a potential target for splicing modulators, we co-transfected the rs1071644-T allele minigene clone into HEK-293 cells in duplicate with splicing factors encoding SRSF1, SRSF2, SRSF5, SRSF6, SRSF7 or, as the negative control, empty pCDM8, as we previously described (Ling & Estus, 2010). After 24 hours, cellular RNA was purified, converted into cDNA, and *D65-PLCG2* and total *PLCG2* quantified as described above for minigene analyses.

### Expression Cloning of *PLCG2* isoforms and Calcium Influx

For expression cloning of *PLCG2* and *D65-PLCG2*, cDNA underwent PCR cycling with primers corresponding to sequences in the *PLCG2 5*’ UTR (5’- CCGATTCCTTCCTTCTCCCTG) and 3’ UTR (5’- CCCAGAGTGTGAATAGGGCA). PCR products corresponding to canonical *PLCG2* and *D65-PLCG2* were separated on a 1% agarose gel, reamplified separately, gel purified and then cloned in-frame at the carboxyl terminus of GFP by using NT-GFP Fusion-TOPO, as directed by the manufacturer (Thermo). Clone identities were confirmed by sequencing (ACGT, Inc). Clones were then transfected into HEK-293 cells by using Lipofectamine 3000 Reagent (Thermo) as directed by the manufacturer. After 24 hours, media was replaced with HBSS with or without 20uM A23187 calcium ionophore (Sigma) for five minutes. Cells were then washed with HBSS, fixed with 10% formalin for 7 minutes, rinsed, and mounted with NucBlueT Fixed Cell Ready Reagent (ThermoFisher). Cells were visualized and representative images obtained by using confocal microscopy (Nikon A1R HD).

### Additional Statistical Analyses

Analyses were performed by using SPSS (V.29). The ratio of different isoforms as a function of genotype was analyzed by Kruskal-Wallis tests. P values for post-hoc analyses were Bonferroni-adjusted for multiple testing.

## 3. Results

**Figure 1:**
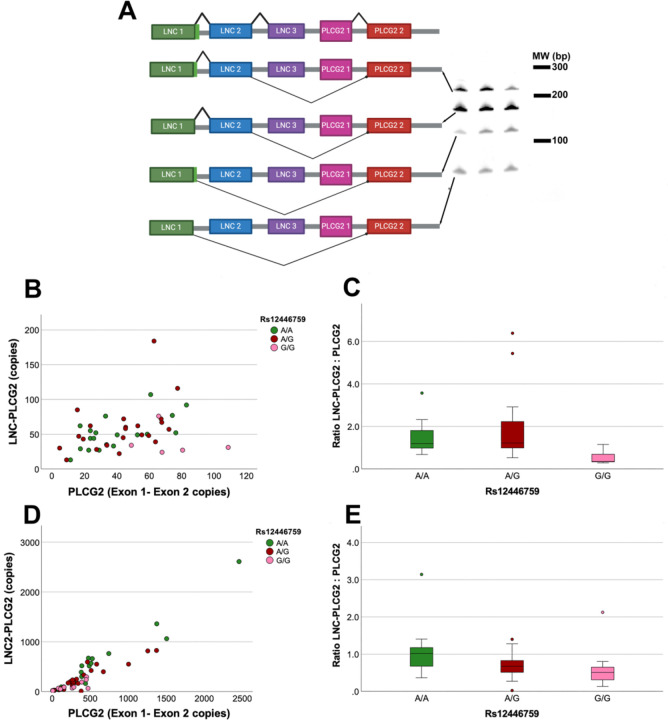
Expression of *LNC-PLCG2* relative to canonical *PLCG2* is associated with rs12446759. Several *PLCG2* isoforms originated with the *LNC* (A). The top row of the graphic shows each of the *LNC* exons and the first two exons of *PLCG2*. The subsequent rows depict each of the variant isoforms that were detected by subjecting human brain cDNA to PCR from *LNCexon* 1 to *PLCG2* exon 2. In addition to the isoform that included *LNC* exons 1 and 2, isoforms were detected that lack *LNC* exon 2, retained 51bp of LNC intron 1 (denoted as light green) and each combination of these possibilities. *LNC-PLCG2* and canonical *PLCG2* were quantified by qPCR in brain (B-C) and buffy coat (D-E) samples. In the brain samples, Kruskal-Wallis analysis found an overall significant association with rs12446759 (p=0.018). Post-hoc analyses found that the homozygous G/G samples were significantly different from the A/G (p=0.022 (Bonferroni-adjusted)) and AA samples (p=0.020). In the buffy coat samples, rs12446759 was also associated with the *LNC-PLCG2*: *PLCG2* ratio (p=0.008, Kruskal-Wallis). Post-hoc analyses found that the homozygous G/G samples were significantly different from the A/A samples (p=0.009). The G/G samples showed a trend for separation from the A/G samples (p=0.076).

As an initial approach to identify *PLCG2* isoforms in human brain, we noted that ENSEMBL (Cunningham et al., 2022) included an isoform wherein a LNC RNA (ENSG00000289733) that flanks *PLCG2* is spliced onto *PLCG2* exon 2, creating a fusion mRNA. Since the first ATG translation initiation site within this fusion mR2, this fusion mRNA still encodes prototypic PLCG2. To check whether this isoform was expr NA is the canonical *PLCG2* translation start site within exon essed in human brain, we performed PCR from the novel LNC exon 1 to exon 2 of *PLCG2*. When these products were visualized by PAGE, four isoforms were detected that were readily detectable in each sample examined (Fig. 1A). Subsequent sequencing of these PCR products found that the most abundant isoform consisted of canonical *LNC* exon 1 and exon 2 that was spliced onto *PLCG2* exon 2 (Fig. 1A). We also detected several less abundant isoforms which sequencing established as (i) *LNC* exon 1 spliced directly onto *PLCG2* exon 2, (ii) *LNC* exon 1 that retained 51 bp intron 1 and then spliced onto *LNC* exon 2 and *PLCG2* exon 2, and (iii) *LNC* exon 1 that retained 51 bp of intron 1 that was spliced directly onto *PLCG2*exon 2 (Fig. 1A).

We proceeded to compare *PLCG2* that arose from the *LNC* versus the canonical *PLCG2* exon 1 by using qPCR on cDNA samples generated from AD and non-AD brains. For this effort, we used a reverse primer in *PLCG2* exon 2 and forward primers in either *PLCG2* exon 1 or *LNC* exon 2 to capture the most abundant novel isoform. This effort revealed that levels of the canonical *PLCG2* and *LNC-PLCG2* isoforms were comparable and were associated with the AD GWAS SNP rs12446759 (Fig. 1B–C). The minor rs12446759-A allele was associated with an increase in *LNC-PLCG2*, compared to the canonical *PLCG2* isoform. To test whether this finding was specific to the brain or relevant to *PLCG2* expression in the periphery, we extended these findings by examining RNA purified from human buffy coat samples. The minor rs12446759-A allele was again associated with an increase in the ratio of *LNC-PLCG2* compared to canonical *PLCG2* (Fig. 1D–E).

We proceeded to evaluate human brain cDNA samples for atypical *PLCG2* splicing. For this effort, we used PCR with primers that generated overlapping fragments of *PLCG2*. Hence, we used primers that corresponded to sequences in exons 1 and 6, exons 5 and 10, exons 9 and 14, exons 13 and 17, exons 16 and 19, exons 18 and 22, exons 20 and 26, exons 25 and 29, exons 28 and 32, and exons 31 and 33.

**Figure 2:**
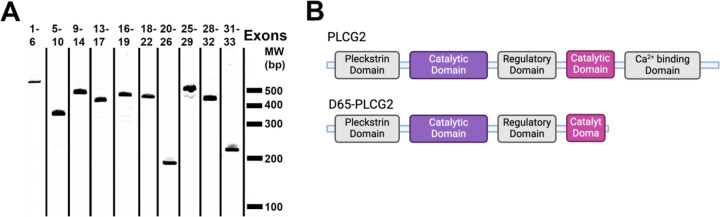
PCR amplification from exons 25 and 29 reveals a novel *PLCG2* variant isoform. PCR was performed on a pool of brain cDNA by using primers corresponding to sequence within the indicated exons (A). The primary novel PCR product, produced by primers against sequences in exon 25 and 29, was found to lack 65bp from the beginning of exon 28. Loss of this 65 bp results in a frameshift with single amino acid followed by premature stop codon (B). This changes *PLCG2* by truncating the second portion of its catalytic domain and deleting the Ca^+2^ binding domain.

The resulting PCR products were the expected sizes except for a product that appeared relatively common and was produced by the primers amplifying from exon 25 to exon 29 (Fig. 2A). This band as well as the expected PCR product were excised from the gel. Sequencing confirmed the larger PCR product was canonical *PLCG2* from exon 25 to 29 and found that the smaller PCR product lacked the initial 65 bp of exon 28 (referred to as *D65-PLCG2*). The use of the atypical splice acceptor site within exon 28 is not unexpected because the underlying RNA sequence (GCACGGGCUACGUUCUGCAG) has a splice acceptor site score of 4.1 which is well above the threshold of 2.2 (Wang & Marin, 2006). That noted, this value of 4.1 is well below the score of 11.2 attained by the canonical splice acceptor site of intron 27 (gcguucacuuuccuucccag) (Wang & Marin, 2006). Regarding the impact of the 65 bp deletion on *PLCG2* protein, this deletion causes a codon reading frameshift such that exon 28 encodes a single amino acid followed immediately by a termination codon. The resulting protein is predicted to lack the carboxyl-terminal 247 amino acids of *PLCG2* (Fig. 2B) which includes the carboxyl portion of the enzymatic Y-box and the C-2 motif that mediates Ca^2+^ binding (Fig. 2B).

**Figure 3:**
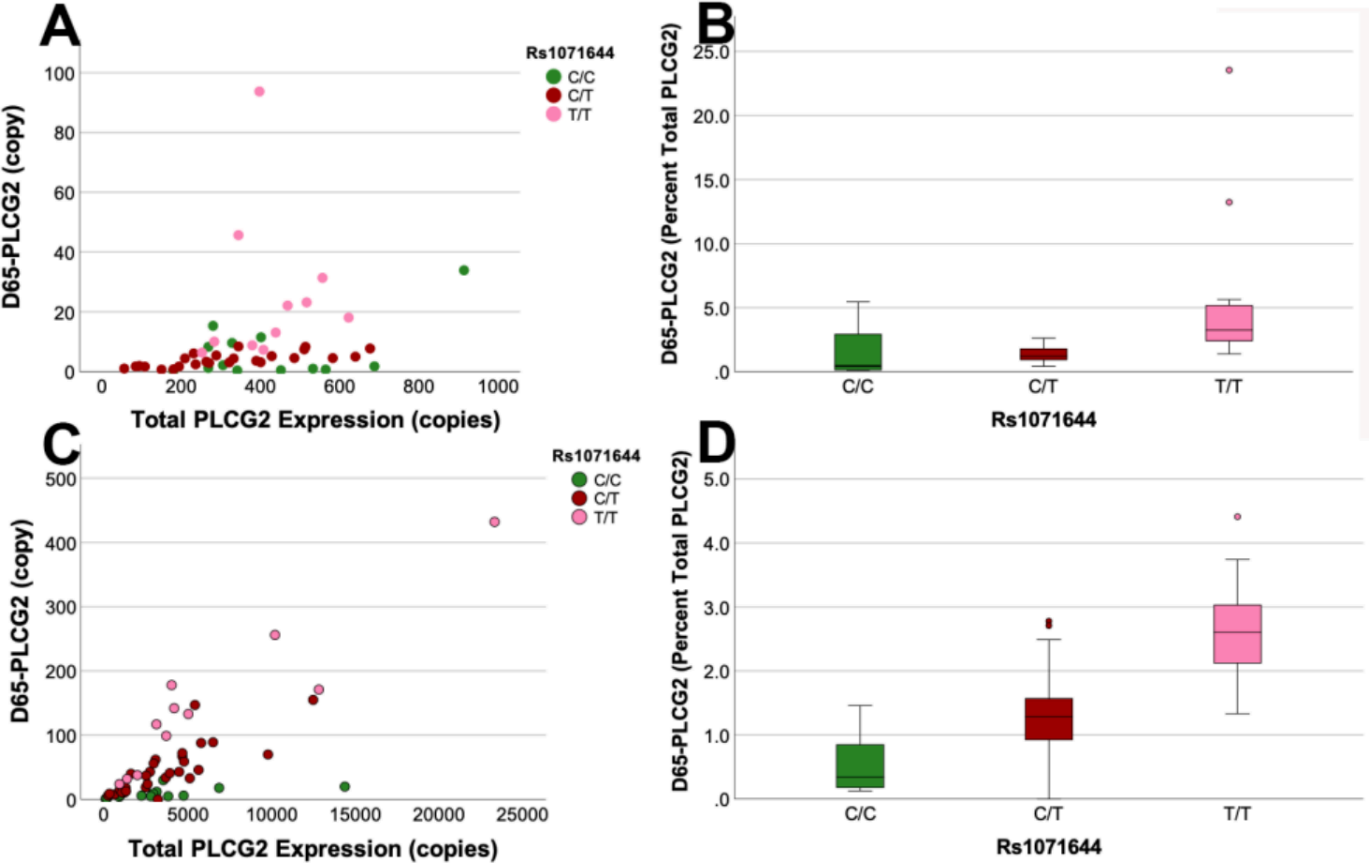
*D65-PLCG2* is strongly associated with rs1071644. This SNP in the skipped portion of exon 28 is a splicing QTL. In the brain samples, Kruskal-Wallis analysis found an overall significant association between the percentage of *PLCG2* expressed as *D65-PLCG2* and rs1071644 (p=2.4×10^−4^). Post-hoc analyses found that the homozygous T/T samples were significantly different from the C/T (p=0.001 (Bonferroni-adjusted)) and C/C samples (p=0.001). In the buffy coat samples, rs1071644 was also associated with the percentage of *PLCG2* expressed as *D65-PLCG2* (p=9.0×10^−7^, Kruskal-Wallis). Post-hoc analyses found that the homozygous T/T samples were significantly different from the C/T samples (p=0.005) and the C/C samples (p=1.4×10^−7^) and that C/T were significantly different from C/C samples (p=0.006).

During this process, we noted that rs1071644 is at position 41 in exon 28, within the skipped 65bp portion. Moreover, DeepCLIP *in-silico* analyses (Gronning et al., 2020) predict that rs1071644 affects binding of splicing factors such as SRSF7; the C allele is predicted to be targeted by SRSF7 (binding score of 0.73) relative to the T allele (binding score of 0.26), and SRSF7 is well-expressed in microglia (Manning & Cooper, 2017). This suggested that rs1071644 may be a functional SNP that influences the proportion of *PLCG2* expressed as *D65-PLCG2*. To evaluate this possibility, we quantified *PLCG2* and *D65-PLCG2* as a function of rs1071644. This qPCR study used a common reverse primer corresponding to sequence within exon 29 and forward primers corresponding to either a constitutively present portion of exon 28 or the unique exon junction generated by exon 27 splicing onto the middle of exon 28. We found that the percent of *PLCG2* that was present as *D65-PLCG2* strongly correlated with rs1071644 in both brain and buffy coat samples (Fig. 3A–D). The proportion of *PLCG2* expressed as *D65-PLCG2* was not correlated with AD neuropathology or sex (p > 0.5).

**Figure 4:**
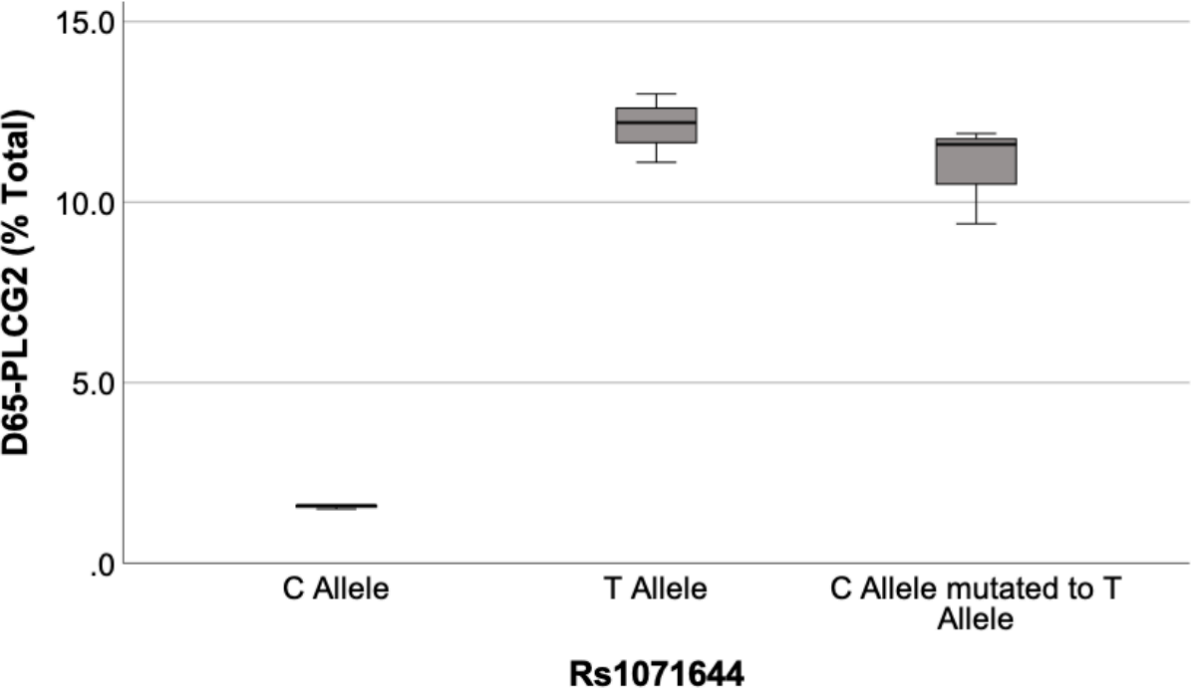
**The proportion of *PLCG2* minigene spliced as *D65-PLCG2* is increased with the T allele of rs1071644 (p<0.001, ANOVA).** The two T clones were not significantly different (p>0.05) but were significantly different from the C allele (p<0.00001). Similar results were observed in a separate experiment in BV-2 cells as well as in HEK293 cells.

To determine whether rs1071644 is indeed a functional SNP that influences skipping of the first 65 bp of exon 28, we cloned minigenes for the rs1071644 major T and minor C alleles that contained *PLCG2* exon 27 to 29, including their respective introns. To obviate the possibility that rs1071644 is a proxy for a different functional SNP that is co-inherited, we mutated the rs1071644 minor C allele clone to the major T allele by using site directed mutagenesis. These three clones were then transfected into murine BV-2 microglial cells. After 24 hours, RNA was prepared, converted into cDNA, and the percentage of *PLCG2* expressed as *D65-PLCG2* was quantified by using a vector specific reverse primer and forward primers corresponding to either the constitutive portion of exon 28 or the novel splice junction formed in D65-PLCG2. We found that the percentage of *D65-PLCG2* arising from the C and T allele clones was 1.4% and 11.1%, respectively, while the samples wherein the minor C allele was mutated to T showed 10.0% *D65-PLCG2* (p = 2×10^−5^, F_1,2_ = 108.4, Fig. 4). Hence, rs1071644 is a functional SNP that affects splicing of *PLCG2* exon 28.

Since rs1071644 is a functional SNP and *PLCG2* has been previously implicated in AD by genetics (Bellenguez et al., 2022; Dalmasso et al., 2019; Jansen et al., 2019; Kleineidam et al., 2020; Magno et al., 2019; Olive et al., 2020; Sims et al., 2017; Strickland et al., 2020; van der Lee et al., 2019), we tested whether rs1071644 is associated with AD risk. Since SNPs are often co-inherited with other SNPs, we first checked whether rs1071644 was in linkage disequilibrium with either of the known AD-associated *PLCG2* SNPs, the rare missense SNP, rs72824905 (P522R) and the GWAS SNP rs12446759. Within the European population that constitutes the samples used within this study, rs1071644 is not co-inherited with rs12446759 (r^2^ = 0.0005, p = 0.758) and only modestly co-inherited with rs72824905 (r^2^ = 0.0205, p = 0.044) (Machiela & Chanock, 2015). To examine the association of rs1071644 with AD further, we evaluated rs1071644 for association with AD by itself and in combination with P522R and rs12446759. We found that rs1071644-T was consistently associated with increased AD risk, independent of the other SNPs (Table 1).

**Figure 5:**
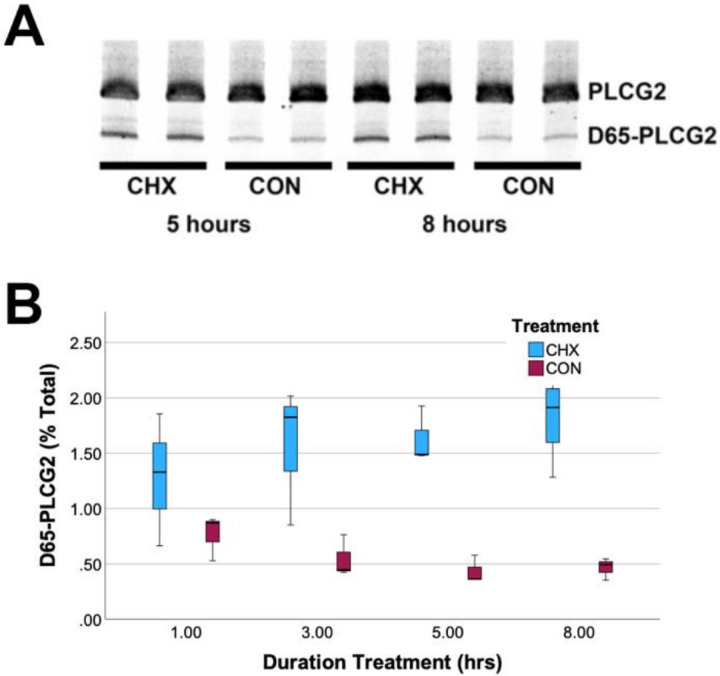
*D65-PLCG2* is subject to NMD. The proportion of *PLCG2* expressed as *D65-PLCG2* is increased in U937 cells treated with CHX, as revealed by visual inspection of PCR products separated by PAGE (A) and by qPCR (B, F_1,3_ = 44.9, p=1.2×10^−6^). CHX treated samples were significantly different from solvent control samples at the 3, 5 and 8 hour time points (P<0.005, LSD).

Since *D65-PLCG2* introduces a premature stop codon within exon 28, well before the usual termination codon in exon 33, we hypothesized that *D65-PLCG2* was susceptible to NMD because this process often occurs when a ribosome encounters a termination codon upstream of an exon junction complex (Silva & Romao, 2009). To test this hypothesis, U937 cells, which naturally express *PLCG2*, were treated with CHX. We and others have previously used CHX to inhibit protein synthesis and, thereby, NMD (Busi & Cresteil, 2005; Malik et al., 2015; Urano et al., 2012; Zajac et al., 2023). RNA was then prepared and reverse transcribed into cDNA. The percentage of *PLCG2* expressed as *D65-PLCG2* was visualized via staining on a polyacrylamide gel and quantified by using qPCR. We found that CHX increased the percentage of *PLCG2* expressed as *D65-PLCG2* (Fig. 5), supporting the hypothesis that *D65-PLCG2* undergoes NMD. Hence, *D65-PLCG2* likely represents a larger proportion of *PLCG2* transcription than suggested by the steady state mRNA measurements.

**Figure 6:**
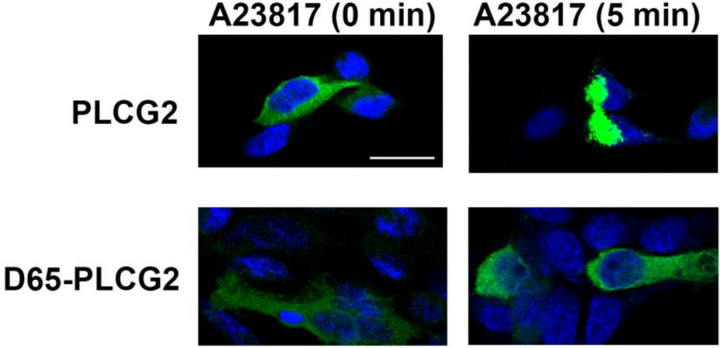
D65-PLCG2 and PLCG2 show cytosolic localization in untreated cells but only PLCG2 becomes condensed after A23187 treatment. Similar results were observed in a separate set of cells.

To discern whether *D65-PLCG2* may encode a stable protein, we generated plasmids encoding *PLCG2* and *D65-PLCG2* as GFP fusion proteins, similar to the approach of Nishida et al who evaluated the role of *PLCG2* domains in *PLCG2* function (Nishida et al., 2003). Clones were transiently transfected into HEK293 cells which typically express PLCG2. In resting cells, the subcellular localization of canonical *PLCG2* and PLCG2-GFP was similar as both manifested a diffuse cytosolic localization (Fig. 6), suggesting that *D65-PLCG2* may be expressed as a stable protein. Since Nishida et al reported that the Ca^+ 2^ binding domain is critical for *PLCG2* response to Ca^+ 2^ (Nishida et al., 2003), we tested the response in cells treated with the Ca^+ 2^ ionophore A23187 for five minutes. While *D65-PLCG2* remained diffusely localized in the cytosol in response to this treatment, *PLCG2* showed a pattern of marked condensation, likely reflecting a conformation change (Fig. 6). Hence, *PLCG2* but not *D65-PLCG2* responds to increases in cytosolic Ca^+ 2^.

**Figure 7:**
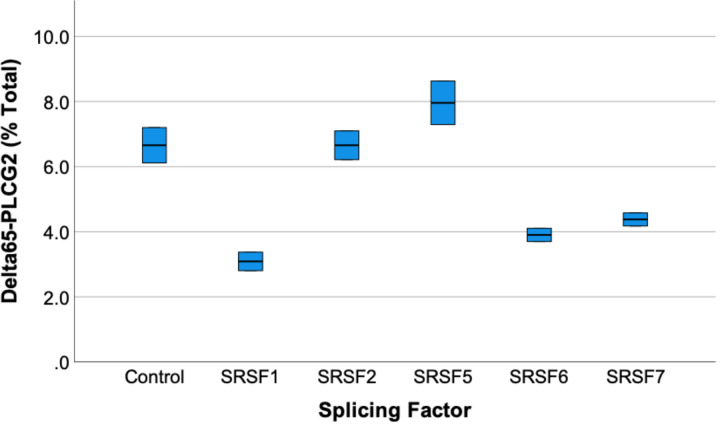
**Several splicing factors, including SRSF7, reduce the proportion of aberrant *PLCG2* exon 28 splicing**(general linear model, p<0.001, results for SRSF1, SRSF6 and SRSF7 significantly different from empty vector control (p<0.01, LSD).

Since rs1071644-T is associated with increased AD risk and causes an increase in *D65-PLCG2*, agents that promote canonical *PLCG2* exon 28 splicing may reduce AD risk. Exon splicing is modulated by splicing enhancer and suppressor proteins that bind to sequence-specific elements within exons and introns and signal to other proteins involved in the splicing process (reviewed in (Manning & Cooper, 2017)). In recent years, pharmacologic agents targeting these splicing factors or the splice sites themselves have emerged as a viable therapeutic strategy (El Marabti & Abdel-Wahab, 2021). As a proof-of-concept study to investigate whether *D65-PLCG23* could be reduced by modulators, the rs1071644-T allele minigene was co-transfected with plasmids encoding splicing modulators, as we and others have described previously (Ling & Estus, 2010; van Bergeijk et al., 2019). These splicing factors included SRSF7, which was noted above to target the rs1071644 sequence, as well as a negative control plasmid. We found that the proportion of *D65-PLCG2* was reduced by ectopic expression of SRSF7, as well as SRSF1 and SRSF6 (Fig. 7), leading us to predict that SRSF1 and SRSF6 bind to other target elements within this minigene. Overall, these results support the concept that *PLCG2* exon 28 splicing can be improved through splicing factor modulation.

## 4. Discussion

This primary findings of this study include (i) a *PLCG2* GWAS SNP, rs12446759, is associated with the proportion of *PLCG2* transcripts that begin within an adjacent LNC versus canonical *PLCG2* exon 1, (ii) levels of a novel *PLCG2* isoform, which lacks the first 65bp of exon 28, are modulated by a SNP within the skipped sequence, rs1071644, that is itself associated with AD risk independent of rs12446759 and P522R, (iii) *D65-PLCG2* is subject to NMD, suggesting that the *D65-PLCG2* mRNA measurements in brain and buffy coat samples likely underestimate the proportion of *PLCG2* expressed as *D65-PLCG2*, (iv) ectopic expression of *D65-PLCG2* as a GFP fusion protein found that *D65-PLCG2* is localized to the cytosol but lacks a Ca^+ 2^ response observed in PLCG2-GFP, and (v) the proportion of *PLCG2* expressed as *D65-PLCG2* is reduced by ectopic splicing factor expression, suggesting that this splicing event may be targetable by pharmacologic agents. In summary, we report mechanisms whereby two SNPs alter *PLCG2* expression and AD risk, test whether a novel isoform is expressed as protein, and show that *PLCG2*splicing can be modulated in a fashion that would reduce AD risk.

The rs12446759-G allele, previously associated with reduced AD risk (Bellenguez et al., 2022), was associated here with a decrease in *LNC-PLCG2* relative to canonical *PLCG2*. Interestingly, rs12446759 is located within the first intron of LNC, 13bp after exon 1, and is therefore contained within the rare *LNC-PLCG2* isoforms that retained 51 bp of *LNC* intron 1. Iin considering how the association of rs12446759 with *LNC-PLCG2* versus *PLCG2* may impact *PLCG2* function, we note that both *LNC-PLCG2* and canonical *PLCG2* encode the same *PLCG2* protein because the first ATG translation start site for either *LNC-PLCG2* or *PLCG2* is within exon 2 of canonical PLCG2. This was also true for each of the multiple *LNC-PLCG2* isoforms that we identified; the first ATG translation start codon for each of isoform is in the canonical *PLCG2* exon 2. We speculate that differences in the 5’UTR sequences of *LNC-PLCG2* and canonical *PLCG2* may influence protein translation. Further work is required to clarify this possibility.

The rs1071644-T allele was associated with an increase in the proportion of *PLCG2* expressed as *D65-PLCG2* in both brain and buffy coat samples (Fig. 3). To test whether rs1071644 may be functional, we compared splicing from minigenes that differed for the rs1071644 alleles. This effort found that rs1071644-T resulted in about 11% of the minigene being expressed as *D65-PLCG2* compared to about 1% for the rs1071644-C allele. Since these minigenes also differed at other SNPs, including rs4611451, rs12596299, rs4888191, rs488192 and rs4369659, we tested whether rs1071644 was indeed functional by converting the rs1071644-C minigene to the T allele by using site-directed mutagenesis. This change was sufficient to change splicing from 1% *D65-PLCG2* to 11% *D65-PLCG2*, replicating the effect of the rs1071644-T allele minigene (Fig. 4). Hence, rs1071644-T increases *D65-PLCG2* and is a functional SNP. Since *D65-PLCG2* introduces a premature stop codon within exon 28, well before the usual termination codon in exon 33, we hypothesized that *D65-PLCG2* was susceptible to NMD because this process commonly occurs when a ribosome encounters a termination codon upstream of an exon junction complex (Silva & Romao, 2009). Consistent with this hypothesis, CHX treated U937 cells showed an increase in the proportion of *PLCG2* expressed as D65-PLCG2. We interpret the finding that *D65-PLCG2* is less stable than *PLCG2* to mean that our measurements of *D65-PLCG2* at steady state in brain and buffy coat likely reflect an underestimate of the proportion of *PLCG2* transcripts that become D65-*PLCG2* during RNA splicing. Hence, the rs1071644-T allele may have a larger effect on overall *PLCG2* transcripts than suggested by our qPCR findings.

Our overall findings support a model wherein the rs1071644-T allele increases AD risk by increasing D65-*PLCG2* at the expense of canonical PLCG2. Since *D65-PLCG2* lacks a portion of the *PLCG2* catalytic domain, and the entire Ca^+ 2^ binding domain, this protein is likely non-functional, as reported previously for a *PLCG2* synthetic construct lacking the Ca^+ 2^ binding domain (Nishida et al., 2003). Because pharmacologic agents have been developed to rectify aberrant splicing and have achieved FDA approval (El Marabti & Abdel-Wahab, 2021; Matsuo, 2021; Neil & Bisaccia, 2019), we tested whether ectopic expression of several splicing factors would reduce D65-PLCG2. This effort found that SRSF1, SRSF6 and SRSF7 were all capable of ameliorating the effects of rs1071644-T on splicing. Hence, the actions of rs1071644-T in promoting AD risk may be countered by responsive to agents that improve canonical exon 28 splicing.

In summary, we report the actions of an AD GWAS SNP, rs12446759, and identify a novel AD risk factor, rs1071644, and its underlying mechanism. Rs12446759 resides just after the first exon of an LNC adjacent to *PLCG2* and is associated with this LNC being used as the *PLCG2* 5’UTR instead of the canonical *PLCG2* exon 1. Rs1071644 resides in exon 28 and is functional in causing a skipping of the first 65 nucleotides of exon 28. While the effects of the altered 5’UTR require further study to elucidate, the changes in exon 28 splicing produce an apparently stable protein which lacks a portion of the PLCG2 catalytic domain as well as the PLCG2 Ca^+ 2^ binding domain. The effects of rs1071644 appear amenable to agents that target splicing, suggesting that this AD risk factor may be amenable to therapeutic intervention.

## Figures and Tables

**Table 1:** Rs1071644 is associated with AD risk.

Reference Allele	Effect Allele*	Model**	OR (95% CI)	Z Statistic	P value
C	T	rs1071644	1.065 (1.017–1.114)	2.706	0.0068
C	T	rs1071644+P522R	1.067 (1.020–1.117)	2.795	0.0052
C	T	rs1071644+P522R+Rs12446759	1.068 (1.021–1.118)	2.843	0.0045

* Each regression models the count of T alleles of rs1071644, reflecting an additive mode of inheritance.

** Sex, age, and the first 10 PCs are covariates in all the regression models. OR: Odds ratio, CI: 95% Confidence Interval

## Data Availability

All relevant data are included within the manuscript.

## Abbreviations

AD: Alzheimer’s disease
MCI: mild cognitive impairment
bp: basepair
LNC: long noncoding
SNP: single nucleotide polymorphism
PIP2: 1-phosphatidyl-1D-myo-inositol 4,5-bisphosphate
IP3: 1D-myo-inositol 1,4,5-trisphosphate
DAG: diacylglycerol
ADGC: Alzheimer’s Disease Genetics Consortium
NACC: National Alzheimer’s Coordinating Center
CHX: cycloheximide
OR: Odds ratio
CI: 95% Confidence Interval
